# A novel ultra-light suction device for mechanical characterization of skin

**DOI:** 10.1371/journal.pone.0201440

**Published:** 2018-08-08

**Authors:** Bettina Müller, Julia Elrod, Marco Pensalfini, Raoul Hopf, Oliver Distler, Clemens Schiestl, Edoardo Mazza

**Affiliations:** 1 Institute for Mechanical Systems, Department of Mechanical and Process Engineering, ETH Zurich, Zurich, Switzerland; 2 Department of Surgery, University Children’s Hospital Zurich, Zurich, Switzerland; 3 Department of Rheumatology, University Hospital Zurich, Zurich, Switzerland; 4 Empa, Swiss Federal Laboratories for Materials Science and Technology, Dübendorf, Switzerland; University of Illinois at Urbana-Champaign, UNITED STATES

## Abstract

Suction experiments have been extensively applied for skin characterization. In these tests the deformation behavior of superficial tissue layers determines the elevation of the skin surface observed when a predefined negative (suction) pressure history is applied. The ability of such measurements to differentiate between skin conditions is limited by the variability of the elevation response observed in repeated experiments. The scatter was shown to be associated with the force exerted by the observer when holding the instrument against the skin. We have developed a novel suction device and a measurement procedure aiming at a tighter control of mechanical boundary conditions during the experiments. The new device weighs only 3.5 g and thus allows to minimize the force applied on the skin during the test. In this way, it is possible to reliably characterize the mechanical response of skin, also in case of low values of suction pressure and deformation. The influence of the contact force is analyzed through experiments on skin and synthetic materials, and rationalized based on corresponding finite element calculations. A comparative study, involving measurements on four body locations in two subjects by three observers, showed the good performance of the new procedure, specific advantages, and limitations with respect to the *Cutometer*^*®*^, i.e. the suction device most widely applied for skin characterization. As a byproduct of the present investigation, a correction procedure is proposed for the *Cutometer* measurements, which allows to partially compensate for the influence of the contact force. The characteristics of the new suction method are discussed in view of future applications for diagnostic purposes.

## 1. Introduction

The mechanical behavior of the skin primarily depends on the connective tissue structures which are present in the epidermis, dermis—consisting of the more superficial papillary dermis and the collagen-rich reticular dermis—and the subcutaneous tissues[1]. The skin, particularly the epidermis and the stratum corneum, functions as a barrier protecting the body from potentially harmful external influence. The characterization of the epidermis is often associated with the development of therapies for pressure ulcers or of cosmetic products[2]. The biomechanical properties of the skin are dominated by the dermis, which consists mostly of collagen (77% of dry weight), elastin (4%), and ground substances[3].

Several skin diseases are associated with structural tissue changes, which lead to abnormal mechanical behavior of the skin. An example is systemic sclerosis (SSc), which is a heterogeneous autoimmune inflammatory disorder characterized by thickening of connective tissues in skin. A progressive fibrotic process and enhanced deposition of collagen results in the disease, leading to significant stiffening of the skin[4,5]. The non-invasive diagnosis of SSc includes a subjective palpation method, so that an objective procedure for characterization of skin biomechanical properties is expected to improve the assessment of disease progression. Another example of potential clinical use of skin biomechanical analysis is for monitoring of healing scars, in particular for cases of large wounds covered by skin grafts. Over the time angiogenesis, continued wound contraction, and eventually connective tissue remodeling might result in the formation of extensive scars. Many ancillary treatment modalities to improve scar quality have been developed, yet the question whether a therapy is indicated for individual patients remains and poses a substantial challenge. This motivates the need for methods to objectively assess scar progression as well as the properties of mature scars[6–8].

In vivo methods for mechanical characterization of superficial skin layers might be considered for such diagnostic applications. They can be grouped in five major classes, including in-situ tensile tests, torsional test, indentation, ballistometry (using the impact of a mass on the skin surface), and tissue elevation methods[9]. The approach most widely used for determining mechanical characteristics of healthy and diseased skin is the suction method[1,2,10–13]. In suction experiments, a negative pressure is applied on the skin surface through an aperture of the suction device. In response to this stimulus, skin tissue is drawn into the probe cavity. The elevation of the skin tissue is quantified and tissue stiffness determined from the ratio of negative pressure and skin displacement. Besides the stiffness, other parameters associated with the viscoelastic and viscoplastic behavior of skin can be determined by controlling the time history of pressure applied in suction experiments. Additionally, different suction openings can be used to observe the mechanical properties of the most superficial layers (2 mm probe aperture) or deeper skin tissues (4 mm– 8 mm) [14,15]. The largest number of skin suction investigations reported in literature used the *Cutometer*^*®*^ *MPA 580* (Courage + Khazaka electronic GmbH, Germany) [16] which was applied to study skin aging[17,18], epidermal hydration[19], systemic sclerosis[13,14,20], and for elasticity measurements in scars[6,21]. Control of pressure increase and decrease rates with the *Cutometer* can be exploited to provide information on skin stiffness, recoverable and dissipative deformation, and viscoelasticity of the skin[1,22]. For measurements on the same skin location, results variability is associated with the compressive force exerted to push the instrument against the skin to ensure contact tightness, as well as possible relative movements between patient and observer during suction. Bonaparte et al. [23] reported the force applied by the observer on the probe, and therefore on the skin, as the most important limitation of suction measurements with the *Cutometer*. Increasing force on the probe resulted in reduction of pliability and elasticity values. Even minor changes in contact force led to significantly different outcomes, affecting interpretation of patient data. Not only inter-rater variability suffers from varying contact forces, but also comparison between studies is difficult. Accordingly, recent investigations introduced specific protocols to minimize the effect of these factors. Weickenmeier et al. [24] and Pensalfini et al. [25] introduced a custom-modified headrest for rigid fixation of participant’s head to prevent variations in probe placement on the face, control of contact pressure and relative movement between probe and measured location. Probably due to limited ability to discriminate between different skin conditions, the *Cutometer* *MPA 580* has never actually become part of standardized applications in clinics.

Based on our experience with suction experiments for soft organs characterization[24–30] we developed a novel suction device and measurement procedure specifically designed to overcome the above limitations for measurements on skin. We call the new device “*Nimble*”. Based on the same principle as the *Cutometer* *MPA 580*, the new device was optimized in terms of weight (down to few grams), in order to avoid the effect of the contact force as well as drastically reduce the influence of patient movement during the measurement. The specific configuration evaluated here was selected such that skin deformation remains relatively low, which was hypothesized to improve the ability of this method to distinguish between skin conditions. The paper presents the new technique; it compares its performance with that of the *Cutometer* when applied on synthetic materials and skin. The influence of contact force is studied through measurements on skin and synthetic materials and corresponding finite element calculations. A procedure is proposed to compensate for this effect in *Cutometer* measurements. Finally, three observers performed suction experiments on four different body locations in two volunteers. Quantitative comparison of the two suction methods includes their ability to distinguish between skin locations as well as the intra- and inter-observer variability. The new suction procedure is shown to offer significant advantages.

## 2. Material and methods

### 2.1 Measurement devices

#### 2.1.1 *Cutometer*

In previous studies, the *Cutometer* was shown to be applicable to various body locations, leading to good reliability, safety, and significance of measurement outcomes[21]. In the *Cutometer* probe a negative pressure is created, drawing the skin into a defined cavity through a circular opening (diameters ranging from 2 to 8 mm). A 6 mm probe opening was selected in order to perform a measurement which includes deeper tissue and not only the most superficial skin layers, as typically achieved with a 2 mm probe opening. In addition, this size has often been applied in previous suction studies on scar tissue [31,32], and this is among the planned future applications of the new device. Inside the probe, skin elevation is determined by a non-contact optical measurement system consisting of a light source and a light receptor, determining the light intensity decrease associated with skin protrusion. Applied pressure history and corresponding elevation curves constitute the main measurement outcome, from which several parameters are extracted. In particular, the parameter R0 (mm) was considered here, which is a measure of the maximum tissue elevation in response to a prescribed negative pressure ramp.

#### 2.1.2 *Nimble*

The novel instrument is named “*Nimble”* and is based on the design of an aspiration device previously applied in clinical studies for the characterization of the uterine cervix[28,33,34]. As described in [35], the new device was minimized in terms of components and features, so to reduce its weight as far as possible. A dedicated set-up and protocol for applications on human skin was realized for the present investigations.

**Fig 1:**
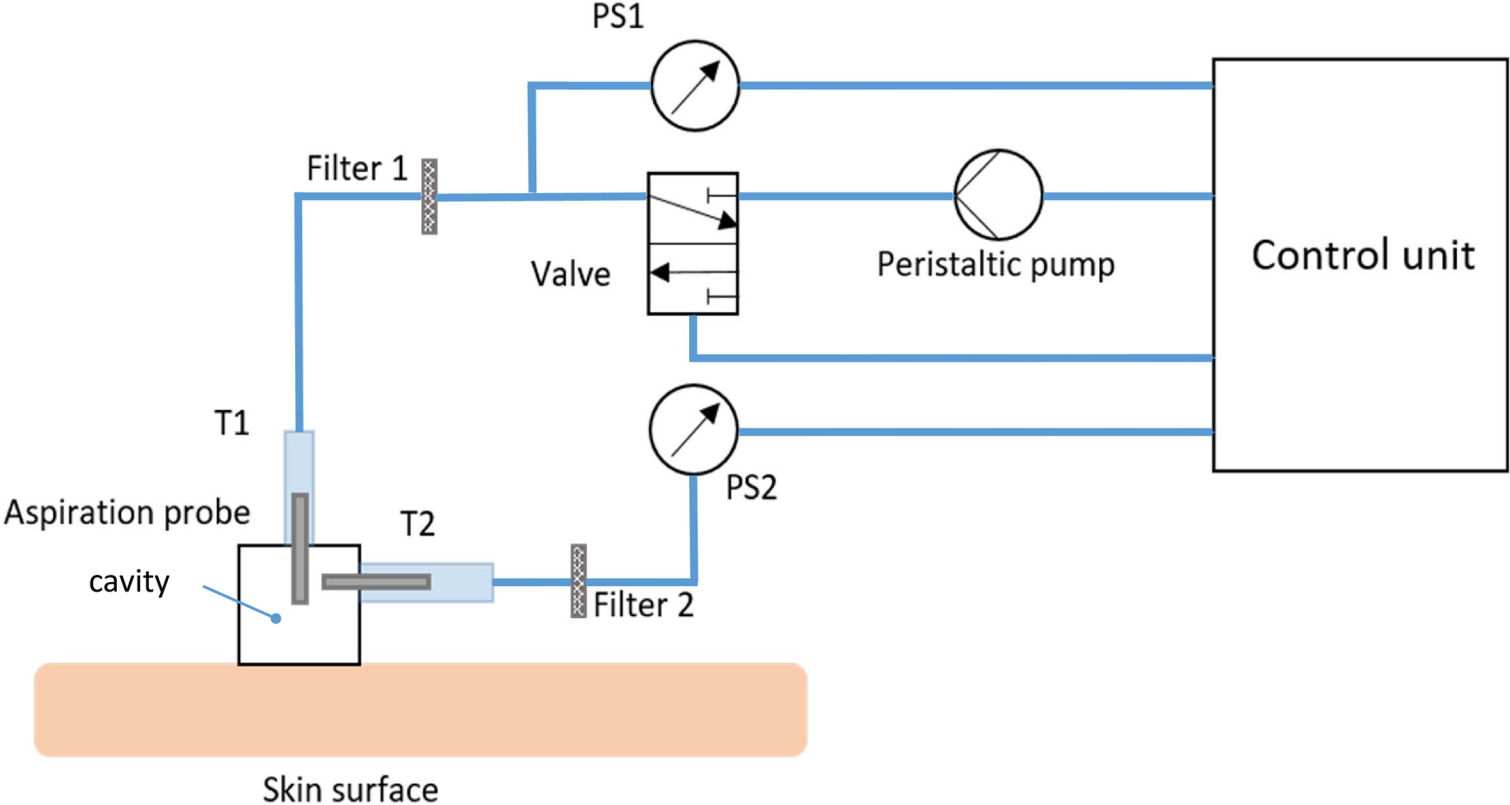
Schematic illustration of the *Nimble* with all components. The aspiration probe is connected (tubes T1 and T2) to pressure sensors PS1 and PS2 via filters 1 and 2. T1 is connected to a peristaltic pump, which generates the progressive negative pressure in the cavity. The valve releases the pressure at the end of the measurement.

**Fig 2:**
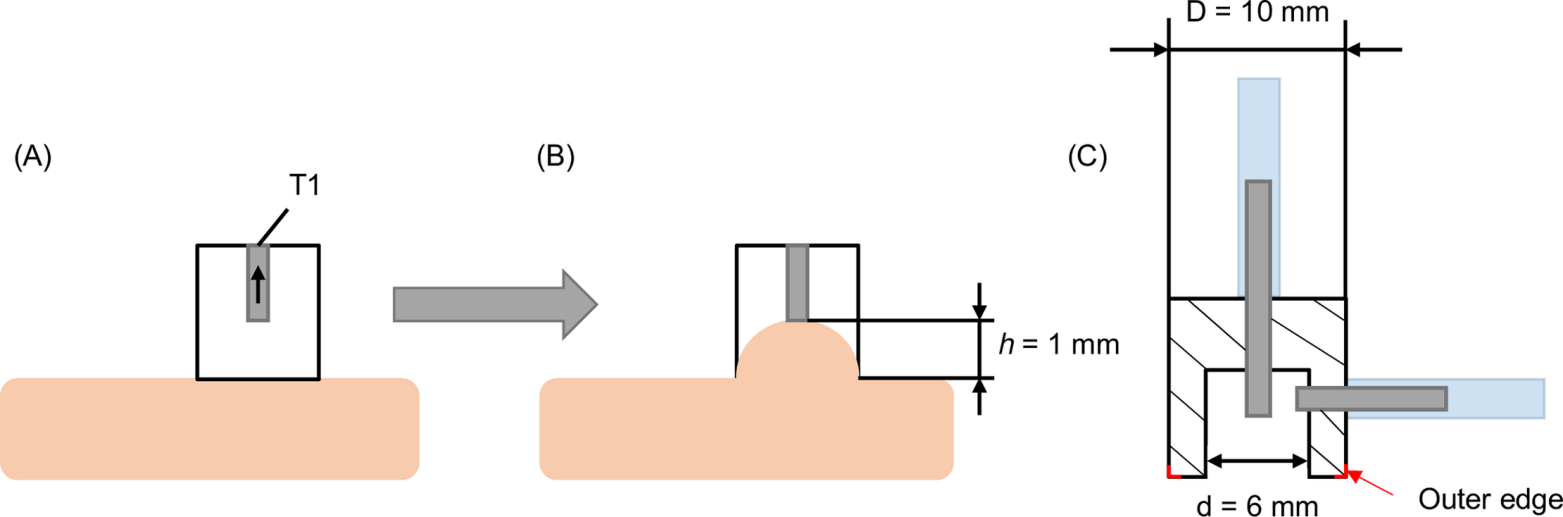
Aspiration probe. (A) Schematic of aspiration probe: The position of the vertical tube T1 defines the elevation height *h*. (B) The tissue is drawn into the cavity until it closes T1. (C) The dimensions of the aspiration probe are indicated.

Fig 1 shows a schematic drawing of the *Nimble*. The main part is the aspiration probe, which is connected to two pressure sensors, *PS1* and *PS2*, via silicone tubes. Between the pressure sensors and the aspiration probe, filters are integrated to avoid contamination associated with airflow. The silicone tubes T1 and T2 are connected to short metal tubes within the measurement probe (Fig 2C). T1 is glued at a distance of *h* = 1 mm from the lower front side (Fig 2B). The pressure tube T1 is connected to the peristaltic pump, which generates a progressive negative pressure within the aspiration probe. As a consequence, skin tissue is sucked into the aspiration probe opening until it reaches a distance *h*. As soon as the tissue touches and seals the tube T1, a difference is detected between the pressure values reported by the pressure sensors PS1 and PS2. Thus, the measurement is automatically stopped and the corresponding closing pressure, *p*_*cl*_ (PS2), is recorded. The valve between *Filter 1* and the peristaltic pump releases the pressure to ensure safe detachment of the probe after the measurement. The probe opening diameter was selected here as d = 6 mm to match the corresponding *Cutometer* characteristic.

The *Nimble* probe is, with Ø 10 mm x 10 mm and a total weight of 3.5 g, smaller and lighter than all existing suction devices, including the lightweight DermaLab USB (Cortex Technologies) and the LASTIC [36]. In particular, when compared with the *Cutometer* (120.8 mm x 26 mm, with a weight of 80 g) the volume and weight of the *Nimble* are more than one order of magnitude smaller.

Different from the *Cutometer*, which measures the elevation obtained for a given suction pressure, the *Nimble* determines the pressure required for a given elevation *h*, i.e. it is a displacement-controlled experiment, which inherently increases the safety of the measurement procedure. The specific value of *h* can be selected for each application. The typical skin elevation obtained in corresponding *Cutometer* measurements ranges between 1.1 and 2 mm [25,37]. For the present study, a value of *h* = 1 mm was selected, thus leading to rather small deformations of the skin in *Nimble* measurements.

The *Nimble* is held by the observer through light and highly flexible silicon tubes and is approached to the skin surface while the peristaltic pump is running. In this way, contact is established without significant forces being exerted on the skin. Due to the lightweight of the probe, the *Nimble* follows possible body movements during the measurements, minimizing possible detrimental influences on the measurement. To facilitate the creation of tight contact between the aspiration probe and the skin surface at the beginning of the measurement, a thin Vaseline layer is applied on the outer edge of the *Nimble*. No Vaseline was required for the *Cutometer* due to the large surface area in contact with skin.

### 2.2 Suction measurement protocol

#### 2.2.1 *Cutometer*

**Fig 3:**
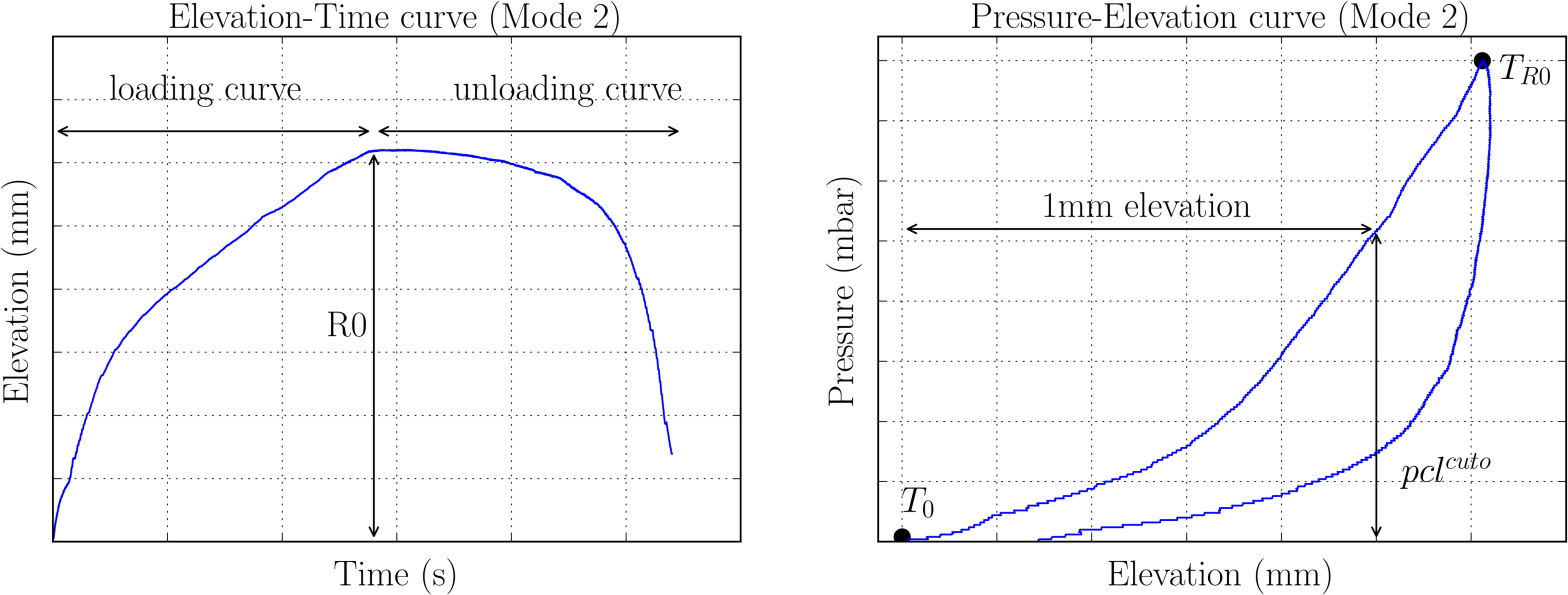
Output curve of *Cutometer* measurements. Representative Elevation-Time curve (left) and Pressure-Elevation curve (right) of a Mode 2 *Cutometer* measurement. The maximum elevation is indicated with R0 and the loading and unloading curves are displayed. From the loading part of the Pressure-Elevation curve the closing pressure at 1 mm elevation is extracted for comparison with the *Nimble* parameter pcl^nimble^. T_0_ and T_R0_ indicate the start of the experiment and the timepoint when R0 is reached respectively. These graphs were generated with a maximum suction pressure of 400 mbar.

The so-called “Mode 2” of the *Cutometer* software was used for this study. A ramp load with 15 mbar/s was applied up to a maximum suction pressure of p_max_ = 400 mbar. Unloading was with the same rate (-15 mbar/s). The Elevation-Time and the Pressure-Elevation curves were extracted for each measurement. Fig 3 shows an Elevation-Time curve, and the maximum elevation of the tissue is the parameter called “R0”, often used in literature[21]. Fig 3 also includes a Pressure-Elevation curve. We used this curve to extract a parameter directly comparable to the closing pressure $p_{cl}^{nimble}$ measured with the *Nimble*: this is the suction pressure needed for a tissue elevation of 1 mm and we call it $p_{cl}^{cuto}$. From both, $p_{cl}^{cuto}$ and R0 we determined corresponding stiffness values (mbar/mm) of the tissue, as $k^{cuto}=\frac{p_{cl}^{cuto}}{1mm}$ and $k^{R0}=\frac{400\mathrm{mbar}}{R0}$ respectively.

#### 2.2.2 *Nimble*

**Fig 4:**
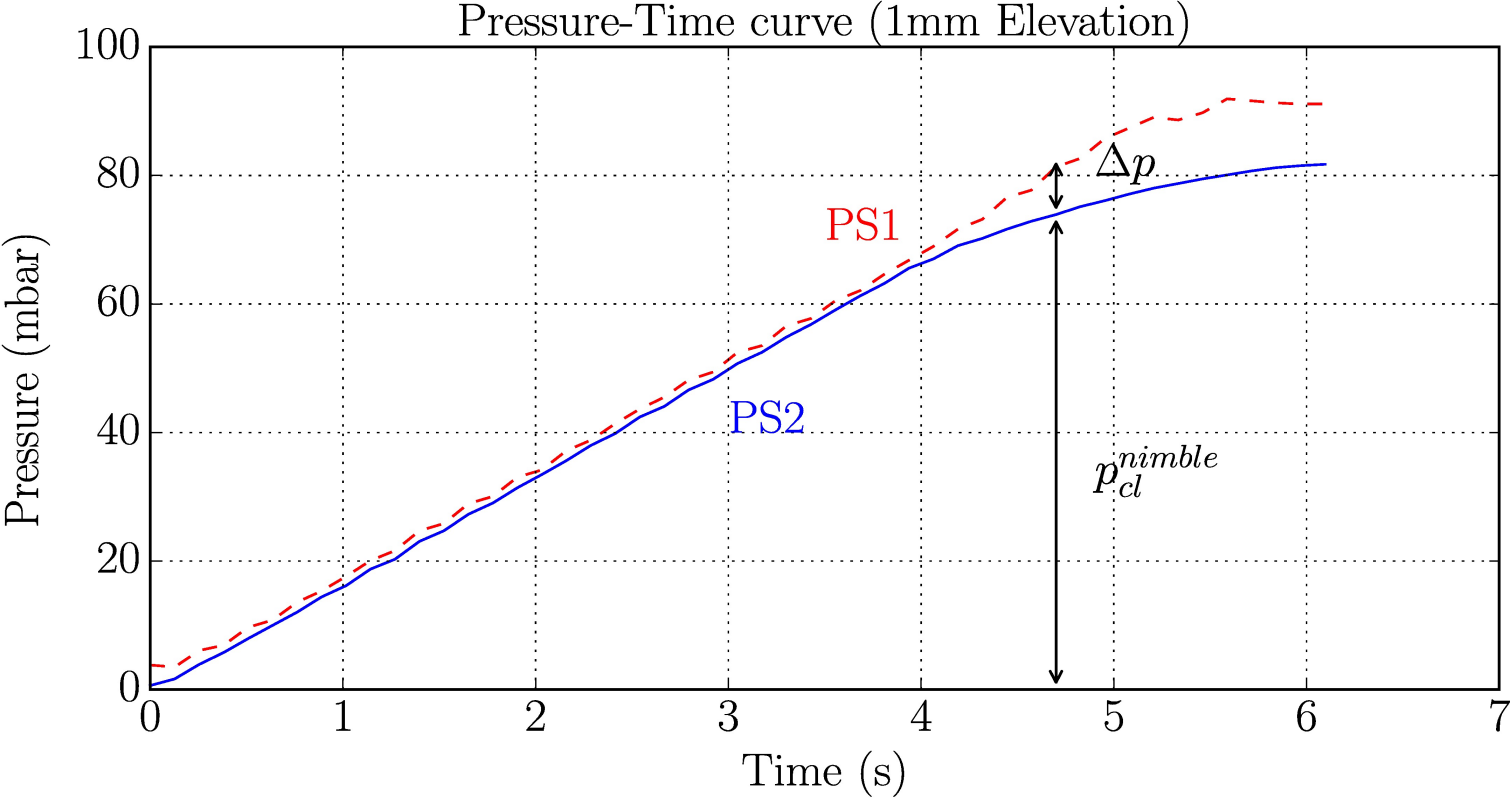
Output curve of *Nimble* measurements. Representative Pressure-Time curve of a *Nimble* measurement for a defined elevation of 1.0 mm. Pressure measured by PS1 and PS2 are indicated. The closing pressure is recorded when Δp = 5 mbar is recognized.

The pressure-loading ramp can be defined through the setting of the peristaltic pump. A rate of 15 mbar/s was selected to match the one of the *Cutometer*. The measurement parameter $p_{cl}^{nimble}$ was recorded for every measurement. Fig 4 demonstrates a Pressure-Time curve of a *Nimble* measurement. The pressure progression measured by the *PS2*, see Fig 1, and *PS1* are indicated. Δp defines the threshold difference (5 mbar) applied to detect the closure of the tube T1. When Δp is reached, the closing pressure $p_{cl}^{nimble}$ is retrieved from the reading of PS1. The stiffness parameter *k* (mbar/mm) for the *Nimble* measurements is calculated as $k^{nimble}=\frac{p_{cl}^{nimble}}{1mm}$.

### 2.3 Preliminary measurements

A two-component silicone elastomer, Ecoflex 0030 (Smooth-On Inc.), was used in this study as a reference material to compare *Nimble* and *Cutometer*. The samples were prepared based on the protocol introduced in Bernardi et al. [38], using a mixing ratio of Part A (base polymer) and Part B (crosslinker) of 1:1. Young’s modulus of the elastomer was measured by indentation tests (FemtoTools AG, FT-MTA02). A vertical arrangement (S1 Fig) was used to induce a controlled contact force in every suction measurement, which was equal to the specimen weight. As shown in S1 Fig, the 10 mm elastomer sample was placed on top of the 6 mm diameter opening for the *Cutometer*. A radial overlap of 2 mm was considered sufficient to generate conditions comparable to those of contact with a half space elastomer sample.

**Fig 5:**
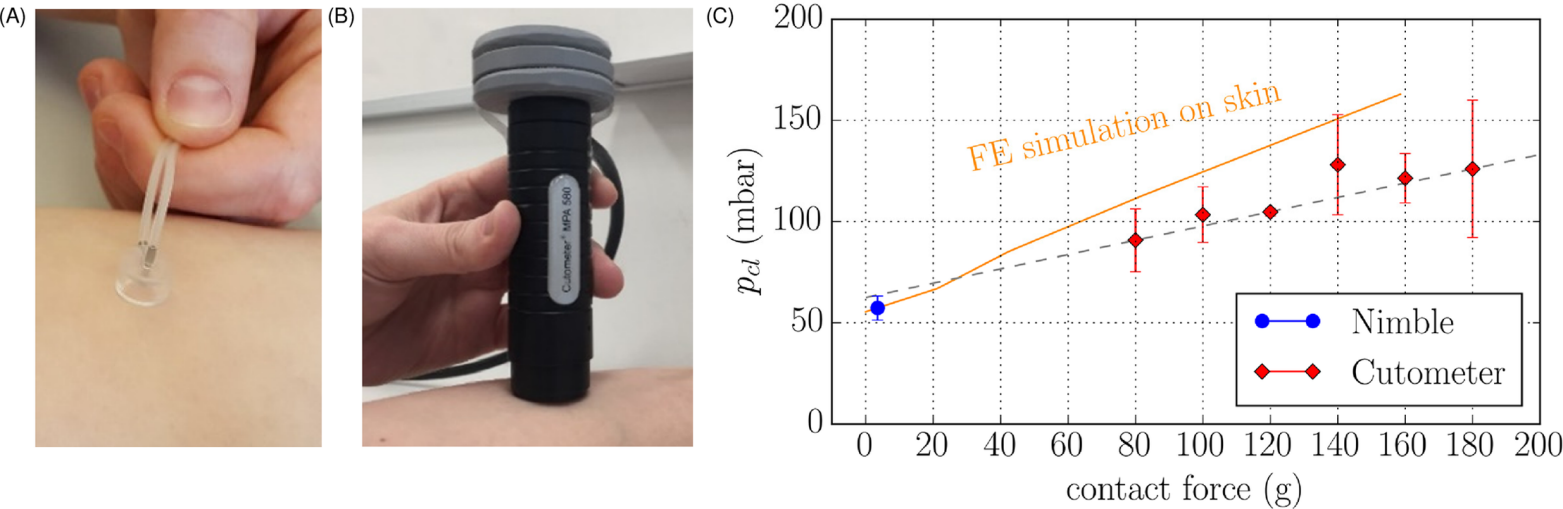
Influence of contact force on volar forearm. Weight study with *Nimble* (A) *and Cutometer* (B) on human volar forearm. The grey blocks indicate added mass of each 20.0 g, corresponding to increased contact force; (C) shows results of measurements on volar forearm with the *Cutometer* (red) and the *Nimble* (blue), and corresponding FE calculations on skin (orange).

The influence of contact force was further analyzed through preliminary measurements on skin. The contact force of the *Nimble* corresponded to its weight (3.5 g) while for the *Cutometer* probe measurements were performed for the probe weight (80 g) as well as for increased force (Fig 5B, grey blocks add a mass of each 20.0 g).

Measurements on elastomers and skin were rationalized with corresponding Finite Element (FE) calculations. S2 Fig shows the corresponding FE meshes and boundary conditions. For the simulation of measurements on skin, the dermis, the subcutaneous tissue and the underlying muscle layer were included in the model as proposed in Weickenmeier et al. [39]. The parameters for the constitutive model of each tissue are reported in S1 Table. For simulations of the measurements on Ecoflex 0030 the model was homogeneous and the elastomer was represented using a Neo-Hookean hyperelastic formulation with the parameter C_10_ = 0.01167 MPa, corresponding to a Young’s modulus E = 6 * C_10_ = 0.07 MPa. More information on the numerical model and the implemented constitutive equations are reported in Weickenmeier et al. [39] and Rubin et al. [40].

### 2.4 Measurements on volunteers

Two volunteers (VO1, VO2, aged between 20 and 30 years) were recruited in summer 2017 at ETH Zurich in agreement with the ethical approval EK 2015-N-63. Signed informed consent was given by each volunteer. Measurements were carried out by three observers (O1, O2, O3) with both devices, *Cutometer* *and Nimble*. The suction measurements were performed on four body locations, namely volar forearm (VF), forehead (FH), back of the hand (BH), and lower back (LB). Body locations were selected in order to test the performance of suction measurements for different conditions and anatomical features of dermis substrates. Each location was measured in triplicates for participant 1 (female) and in sextuples for participant 2 (male). In order to evaluate the influence of the number of measurement repetitions, suction experiments in the second subject were repeated six times. Between every measurement on the same location, a waiting time of at least 45 seconds was ensured, based on prior experience on tissue recovery after suction experiments[25]. A total of 216 suction experiments were performed.

### 2.5 Statistical analysis

For statistical analysis, the Python library scipy.stats was used (Python Software Foundation). The analysis included descriptive statistics, inter alia, calculation of minima and maxima, means (MEAN), standard deviations (STD), and coefficient of variation CV = STD/MEAN. Pairwise correlation of repeated measurements was plotted. Statistical significance of effect of the chosen method, different positions, and multiple observers was assessed with a two-sided t-test (stats.ttest_ind) and the level of significance is indicated for every measurement (p < 0.05 “*”, p < 0.01 “**” and p < 0.001 “***”). The reliability of the devices was examined by means of the intraclass correlation coefficient (ICC), where a class 2 ICC (all observers evaluate all locations) was applied and the absolute agreement calculated. Concurrent validity was assessed between the stiffness measurements of both suction devices by means of a Pearson correlation (stats.pearsonr). The calculation of the root mean square (RMS) is used for analysis of results.

## 3. Results

### 3.1 Preliminary measurements

Measurements on the Ecoflex specimen were performed in order to evaluate the reliability of the Nimble compared to the Cutometer. The repeatability of the measurements is excellent for both devices, and the closing pressure measured with the Nimble is in perfect agreement with the prediction of corresponding FE calculations for m = 0 g contact force (S3 Fig). Larger contact forces lead to a larger pre-deformation with the elastomer protruding more into the probe S1B Fig. As confirmed by corresponding simulations (S2 Table), this pre-deformation reduces the suction pressure (in case of an elastomer) required to reach 1 mm elevation, for both devices.

The difference between *Cutometer* and *Nimble* measurements is statistically significant, with the *Cutometer* reading about 10% higher than *Nimble*. Various factors were analyzed and a series of tests were performed in order to rationalize this difference. We considered the influence of probe geometry, inaccuracies in pressure readings and the influence of friction coefficient. For the latter, a FE based parametric study was performed considering conditions ranging from frictionless to dry friction (leading for both devices to a friction coefficient of μ = 1). The results indicate that these factors can justify the observed systematic difference between *Nimble* and Cutometer, which will be accounted for in the following sections.

The results of the measurements on elastomer indicate that the contact force present during the measurement influences the measured tissue stiffness. Since this effect is expected to depend on the mechanical behavior of the material investigated, the influence of contact force is analyzed for measurements on skin with both *Nimble* and *Cutometer*. We performed tests on the volar forearm of the volunteer VO1, Fig 5.

Results reported in Fig 5 indicate a notable increase in closing pressure with increasing contact force. Interestingly, a linear interpolation through the *Cutometer* data extrapolated down to zero force matches to a great extent the measurement obtained with the *Nimble*. Corresponding FE calculations were performed with constitutive model parameters for skin selected to match the *Nimble* measurement. The prediction obtained using these parameters for increased contact forces qualitatively confirms the experimental observations, with increasing apparent stiffness for larger force values. The corresponding data obtained from measurements on Ecoflex (section 3.1) are reported in the supplementary information (S3 Fig). They show that for an elastomer, the apparent stiffness (slightly) decreases for increasing contact force. This result is in line with corresponding FE calculations and is associated with the difference in strain stiffening behavior between skin and elastomer.

The devices were further analyzed in terms of their sensitivity to patient movements (rotation in the horizontal plane and vertical motion). Results are reported in supplementary information (S4 Fig) and indicate that the effect of skin movement was negligible for the *Nimble* while significant influences were observed for the *Cutometer*.

The results of Fig 5 confirm previous data[23], indicating a notable influence of contact force on suction measurements. While improved measurement protocols can help minimizing this[25], in clinical applications, the contact force applied with a hand—held device is difficult to control. For this reason, a simple correction procedure was developed to compensate (to some extent) for this influence, as described in the next section.

### 3.2 Correction scheme for contact force in *Cutometer* measurements

**Fig 6:**
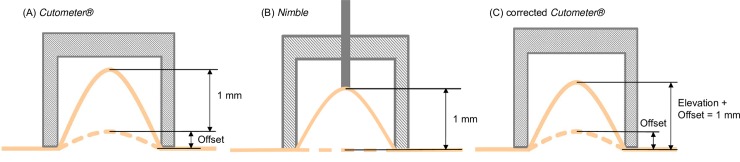
Representation of the correction procedure. (A) Cutometer measures tissue elevation from a baseline, defined by the apex of an initial deformation (response to contact force). (B) Nimble measures tissue elevation from skin surface (negligible initial deformation) due to its low weight. (C) The correction scheme accounts for this discrepancy and adds the Offset to the elevation measured by the Cutometer.

The contact force between suction probe and skin surface causes an initial deformation, leading to an initial tissue protrusion into the suction cavity. This initial deformation is measured by the *Cutometer* and displayed as “Offset”, which is proportional to the force applied by the operator. As illustrated in Fig 6A, the *Cutometer* measures tissue elevation starting from the initial position. Thus, when the software indicates 1 mm protrusion, the total elevation is higher in that the Offset should be added to it. When compared to the situation of the *Nimble* (negligible force and pre-deformation), the actual skin deformation at 1 mm elevation is higher for the *Cutometer*. As an alternative, it is proposed to compare the pressure values for a total elevation of 1 mm of the *Cutometer*, calculated as the reported elevation plus the Offset (Fig 6C).

The proposed correction accounts for the deformation induced by the contact force as if it would be a consequence of suction. In order to analyze the consequences of this simplification, corresponding FE calculations were performed, in which an initial deformation was generated through corresponding contact forces of 50 g and 100 g. The results are summarized in Table 1. The reference values of closing pressure for skin are those calculated for zero initial force. The error associated with the pre-deformation (without correction) is much larger for skin than for the elastomer (S2 Table). The proposed correction scheme provides a realistic estimation of the expected closing pressure. Note that the correction leads to an overestimation of the closing pressure for the skin while it underestimates the stiffness of the elastomer. This confirms that the effect of contact force depends on the constitutive behavior of the examined material and its strain-stiffening or softening response. A generalized correction scheme can therefore not be determined a priori. However, it seems that the proposed correction drastically reduces the effect of contact force for measurements on skin and it is therefore considered for the analysis of the human skin data reported in the next section. Note that contact force fluctuation during the measurements cannot be accounted for by this correction.

**Table 1:** Analysis of the proposed correction scheme for *Cutometer* measurements. Results of FE calculations with enforced initial deformation (corresponding to contact forces of 50 g and 100 g) are compared for closing pressure values before and after correction. Calculations were performed for skin.

	Skin			Skin_corrected	
	0 g	50 g	100 g	50 g	100 g
**PCL (error %)**	55.4 mbar (0.0)	101.7 mbar (83.6)	134.8 mbar (143.4)	56.7 mbar (2.3)	59.8 mbar (8.0)

### 3.3 Measurements on volunteers

Measurement results are summarized in Table 2 and Table 3. Mean values and standard deviation of data obtained by the three independent observers (O1, O2, and O3) on participants VO1 and VO2 at four different body locations (VF, FH, BH, and LB) are reported for three measurements (for VO1) or six measurements (VO2) at each location (complete data shown in S3 Table). The closing pressures obtained with the *Nimble* are generally much lower than those of the *Cutometer*. Note that no correction is applied for these data (see section 3.2). The elevation obtained with the *Cutometer* for 400 mbar suction pressure is much larger than the one applied with the *Nimble* (1 mm). As shown in Table 2 and Table 3, no significant improvement of variability was obtained when repeating the measurements six times (VO2) instead of three times (VO1). A systematic analysis of these data and comparison of the results obtained with the two devices is reported in the next sections.

**Table 2:** Measurements on volunteer VO1. Reported are mean data and standard deviation of repeated measurements by three observer (O1, O2 and O3) on the subject VO1 at four body locations (VF, FH, BH and LB). Data include the Offset Δ, the maximum elevation R0 and the closing pressure $p_{cl}^{cuto}$ of *Cutometer*, and $p_{cl}^{nimble}$ of *Nimble*.

VO1									
		Offset (mm)		pcl^cuto^ (mbar)		R0 (mm)		pcl^nimble^ (mbar)	
		mean	std	mean	std	mean	std	mean	std
**Volar forearm**	**Observer 1**	0.180	0.027	180.00	40.43	1.42	0.11	71.48	6.89
	**Observer 2**	0.341	0.028	140.00	10.71	1.71	0.02	57.44	8.23
	**Observer 3**	0.271	0.039	102.67	18.21	2.15	0.20	55.49	3.59
**Forehead**	**Observer 1**	0.358	0.042	181.00	47.00	1.42	0.19	50.48	6.12
	**Observer 2**	0.616	0.056	344.00	70.73	1.09	0.11	38.26	3.21
	**Observer 3**	0.455	0.055	192.67	38.13	1.28	0.07	29.30	1.80
**Back of hand**	**Observer 1**	0.355	0.036	84.00	28.71	1.72	0.14	32.22	1.17
	**Observer 2**	0.363	0.054	71.33	14.82	1.75	0.16	27.86	1.49
	**Observer 3**	0.358	0.125	87.33	29.32	1.74	0.12	32.53	4.24
**Lower back**	**Observer 1**	0.218	0.055	107.33	3.40	1.80	0.04	70.62	2.84
	**Observer 2**	0.288	0.039	106.67	4.71	1.92	0.07	71.03	4.38
	**Observer 3**	0.343	0.025	121.33	12.68	1.80	0.04	74.19	2.37

**Table 3:** Measurements on volunteer VO2. Reported are mean data and standard deviation of repeated measurements by three observer (O1, O2 and O3) on the subject VO2 at four body locations (VF, FH, BH and LB). Data include the Offset Δ, the maximum elevation R0 and the closing pressure $p_{cl}^{cuto}$ of *Cutometer*, and $p_{cl}^{nimble}$ of *Nimble*.

VO2									
		Offset (mm)		pcl^cuto^ (mbar)		R0 (mm)		pcl^nimble^ (mbar)	
		mean	std	mean	std	mean	std	mean	std
**Volar forearm**	**Observer 1**	0.291	0.064	78.33	12.83	1.90	0.24	51.72	6.14
	**Observer 2**	0.322	0.063	69.33	11.12	2.16	0.21	45.04	6.22
	**Observer 3**	0.655	0.293	139.00	53.28	1.86	0.44	48.01	6.01
**Forehead**	**Observer 1**	0.678	0.103	142.67	19.28	1.57	0.11	15.65	1.37
	**Observer 2**	0.760	0.139	121.33	44.91	1.53	0.11	15.51	1.37
	**Observer 3**	0.888	0.197	176.67	47.15	1.43	0.15	20.59	2.20
**Back of hand**	**Observer 1**	0.299	0.109	55.33	6.90	2.05	0.15	42.65	4.01
	**Observer 2**	0.589	0.153	80.67	19.38	1.93	0.15	35.38	3.05
	**Observer 3**	0.407	0.076	61.67	8.75	2.04	0.13	47.59	5.50
**Lower back**	**Observer 1**	0.254	0.046	87.67	1.80	2.14	0.06	61.25	3.58
	**Observer 2**	0.421	0.041	99.67	3.90	2.08	0.06	56.43	3.32
	**Observer 3**	0.403	0.059	95.00	4.86	2.05	0.05	53.91	4.37

#### 3.3.1 Comparison of mean stiffness values

As a first general comparison of the tissue characterization based on the two instruments, the mean stiffness values of each of the four locations in the two subjects were compared. The corresponding concurrent validity was calculated between *k*^*nimble*^ and the stiffness measured with the *Cutometer* (*k*^*cuto*^ and *k*^*R*0^) by means of a Pearson correlation, see Table 4. In particular, the values -1 or +1 imply a linear relationship with direct and inverse correlation, whereas 0 implies no correlation.

**Table 4:** Pearson correlation coefficient. *r* and p-values, *p*, between stiffness values evaluated with *Nimble* (***k***^***nimble***^) and *Cutometer* (***k***^***cuto***^ and ***k***^***R*0**^).

	Pearson's correlation coefficient					
			k^cuto^	k^cuto^_corr_	k^R0^	k^R0^_corr_
**k**^**nimble**^		r	-0.23	0.66	-0.48	-0.14
		p	2.57e-02	3.02e-13	7.25e-07	1.82e-01

Interestingly, the correlation of *k*^*nimble*^ with *k*^*cuto*^ and *k*^*R*0^ is weak and negative. On the contrary, after application of the correction scheme proposed in section 3.2, a positive correlation is found between *k*^*nimble*^ and $k_{corr}^{cuto}$. The correction does not improve the correlation of the *Nimble* measurements with that based on R0, confirming that, due to the pronounced strain stiffening behavior of skin, the characterization at low values of suction pressure (*k*^*nimble*^) provides a different information with respect to that at higher deformations (*k*^*R*0^).

**Fig 7:**
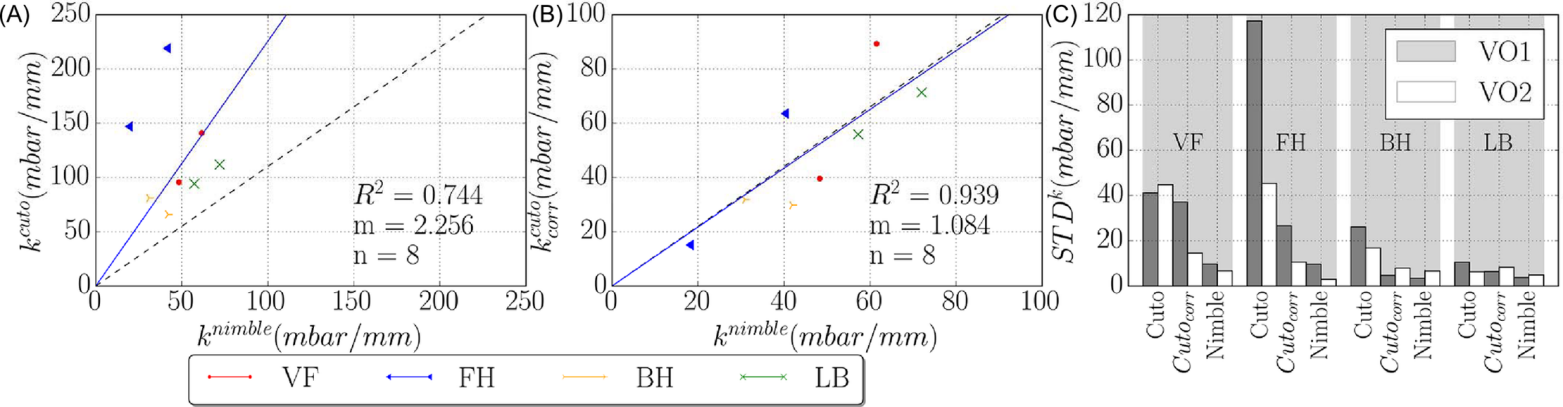
Mean stiffness and standard deviation for each location and subject. (A) and (B): linear regression for a slope of 1.1 between mean stiffness ***k***^***cuto***^ and ***k***^***nimble***^ (A) and corrected *Cutometer* data (B), for eight different skin locations. (C): standard deviation of stiffness measurements for each body location separated for two subjects (VO1 and VO2).

To analyze the correlation between *k*^*nimble*^ with *k*^*cuto*^ and $k_{corr}^{cuto}$ further, Fig 7 shows the analysis of the linear regression between mean stiffness values measured by C*utometer* over mean stiffness values measured by *Nimble*, based on the 1 mm closing pressure. Data points correspond to eight skin locations (four in each volunteer). According to the results of section 3.1, linear regression with linear least squares and zero intercept was analyzed for a slope of 1.1 (see dotted lines in the diagrams), indicating an expectation of 10% higher stiffness values for *Cutometer* measurements compared to *Nimble*. While the comparison with the non-corrected values indicates a much higher stiffness determined with *Cutometer*, after correction the results align reasonably well (Fig 7B). In addition, the correction notably reduces the scatter of the *k*^*cuto*^ values, in particular for the forehead (Fig 7C). These results indicate that the proposed correction scheme can effectively be used to reconciliate *Nimble* and *Cutometer*.

**Fig 8:**
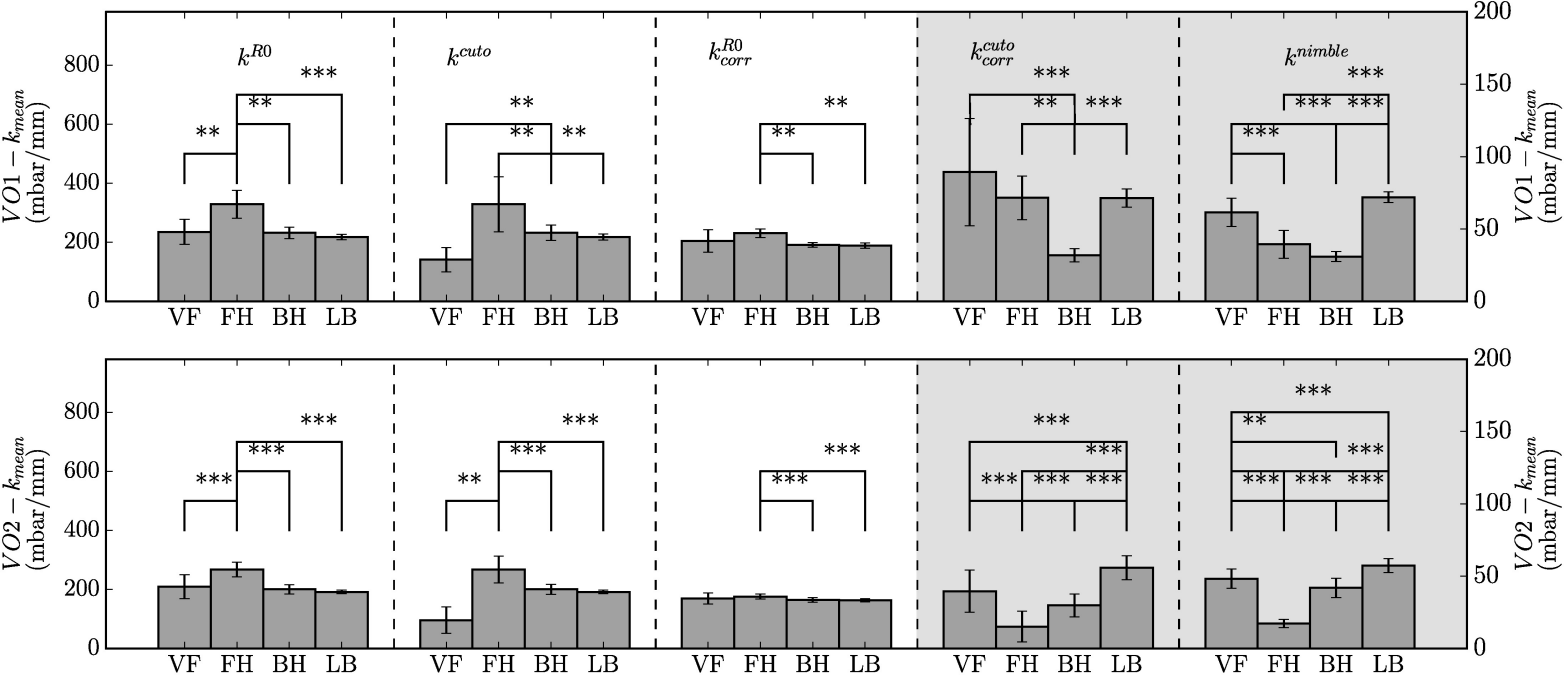
Mean values and standard deviation of stiffness values *k*^*R*0^, *k*^*cuto*^, $k_{corr}^{R0},\;k_{corr}^{cuto}$ and *k*^*nimble*^. Values are shown for each location separated by subjects. Significant difference between locations measured by each device are indicated. Significance level are indicated as **p < 0.01 and *** p < 0.001.

Mean values and standard deviations of stiffness values obtained for the different skin locations in each volunteer were analyzed to evaluate the ability of each parameter to distinguish among different skin conditions. To this end, each location has been considered as a specific specimen for each volunteer, see Fig 8. *Nimble* measurements indicate significant differences for 10 out of 12 inter-specimen comparisons. Corrected $k_{corr}^{cuto}$ values differentiated 8 out of 12 and *k*^*R*0^ 6 inter-specimen comparisons. The consistency between corrected $k_{corr}^{cuto}$ and *k*^*nimble*^ is evident in Fig 8, further confirming the usefulness of the correction. Interestingly, the correction leads to a homogenization of the $k_{corr}^{R0}$ values so that the ability of this parameter to distinguish locations decreases. For the non-corrected values, the *Cutometer* indicates the lower back as the softest location for both volunteers, while it is among the stiffest based on *k*^*nimble*^ as well as corrected $k_{corr}^{cuto}$.

As a next step, each of the four locations of the two subjects was considered as a specimen and a coefficient was calculated which reflects the ability of measurement procedure to differentiate among specimens[41], i.e. the *Intraclass Correlation Coefficient* (ICC) reported in Table 5. It is a class 2 ICC, in which all observers evaluate all specimens, and the absolute agreement is evaluated. In line with the data of Fig 8, the *Nimble* leads to the highest ICC (r_VO1_ = 0.88 and r_VO2_ = 0.94, strong reliability), followed by *k*^*R*0^ the corrected $k_{corr}^{cuto}$. The stiffness evaluation based on $p_{cl}^{cuto}$ is less reliable and the stiffness based on R0 does not improve with the correction.

**Table 5:** Reliability of measurements with *Cutometer* (*k*^*R*0^ and *k*^*cuto*^, non-corrected and corrected), and *Nimble* (*k*^*nimble*^). Intraclass correlation coefficient reflects the ability of the parameter to differentiate among specimens in each subject (here: locations) with n_VO1_ = 36 and n_VO2_ = 72.

	k^R0^	k^R0^_corr_	k^cuto^	k^cuto^_corr_	k^nimble^
**ICC**_**VO1**_**(2,1)**	0.64	0.22	0.37	0.58	0.88
**ICC**_**VO2**_**(2,1)**	0.87	0.28	0.62	0.83	0.94

#### 3.3.2 Intraobserver and interobserver variability

**Fig 9:**
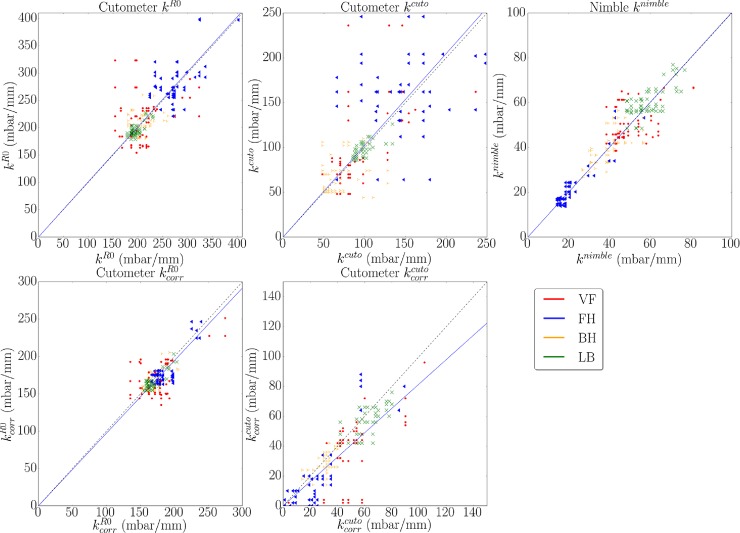
Intraobserver variability in terms of linear dependency. Linear dependency of stiffness values ***k***^***R*0**^**, *k***^***cuto***^, ***k***^***nimble***^, $k_{corr}^{R0}$ and $k_{corr}^{cuto}$ based on repeated measurements at each location (VF, FH, BH and LB).

Based on the repeated measurements performed at each location by each observers (n = 3 for VO1 and n = 6 for VO2), the intraobserver variability was analyzed in terms of stiffness values. Each measurement result is reported against the successive one in the diagrams of Fig 9. Table 6 summarizes the coefficient of determination (R^2^) of the latter for each location. The measurements with the *Nimble* as well as those based on R0 show very low variability at every location (0.964 ≤ R^2^ ≤ 0.991 and 0.934 ≤ R^2^ ≤ 0.998 respectively). Even better is the correlation for corrected $k_{corr}^{R0}$ values (0.982 ≤ R^2^ ≤ 0.998), but note that points tend to cloud indicative of the increased homogeneity of these results (which leads to a lesser ability to distinguish skin conditions, see previous section). The *k*^*cuto*^ measurements show larger variability, with an improvement associated with the correction. As expected, the excellent repeatability of measurements performed on synthetic materials (section 3.1) could not be observed in measurements on skin (CV^nimble^ = 8.68% and CV^cuto^ = 17.93%). These findings indicate that skin is very sensitive to interactions with the observer, and that this effect can be reduced with the *Nimble* due to its lightweight solution.

**Table 6:** Coefficient of determination R^2^. R^2^ of intraobserver variability (in terms of linear regression) in stiffness values *k*^*R*0^, *k*^*cuto*^, *k*^*nimble*^, $k_{corr}^{R0}$ and $k_{corr}^{cuto}$ based on repeated measurements at each location (VF, FH, BH and LB).

**Intraobserver variability**					
	**k**^**R0**^	**k**^**cuto**^	**k**^**nimble**^	**k**^**R0**^_**corr**_	**k**^**cuto**^_**corr**_
**VF**	0.934	0.826	0.964	0.982	0.867
**FH**	0.985	0.876	0.976	0.996	0.799
**BH**	0.986	0.876	0.976	0.996	0.955
**LB**	0.998	0.994	0.991	0.998	0.979

**Fig 10:**
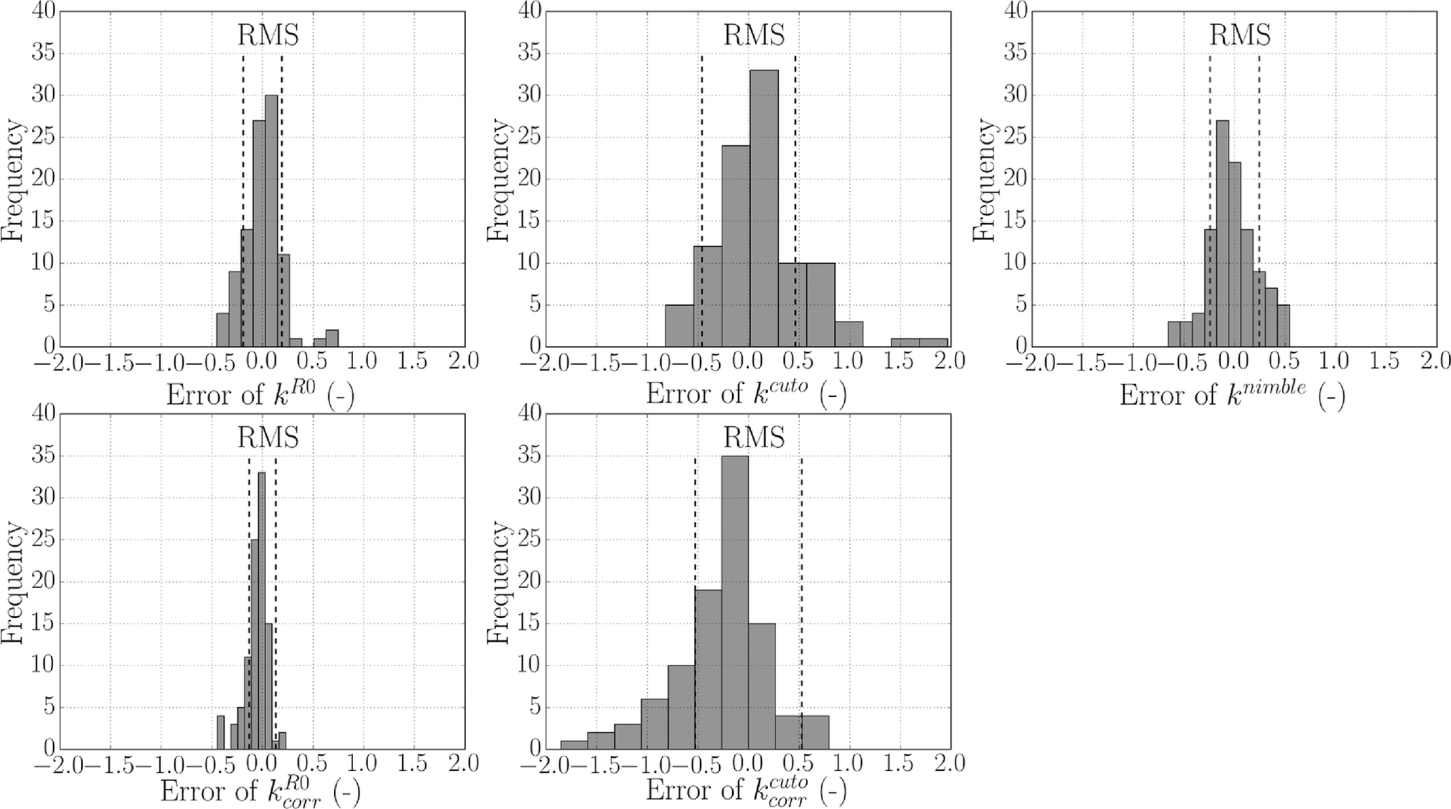
Histogram plot of error between measurements of different observers (n = 3) for stiffness values *k*^*R*0^, *k*^*cuto*^, *k*^*nimble*^, $k_{corr}^{R0}$ and $k_{corr}^{cuto}$. The error is defined as the percentage difference of each measured parameter with respect to the mean value for that sample. Root mean square (RMS) is indicated for each parameter (***RMS***^***R*0**^ = 19%, ***RMS***^***cuto***^ = 46%, ***RMS***^***nimble***^ = 24%, ${RMS}_{corr}^{R0}$ = 13% and ${RMS}_{corr}^{cuto}$ = 53%).

The error between measurements of different observers is defined as the percentage difference of each measured parameter with respect to the mean value for that location. Corresponding histogram plots are shown in Fig 10 (bin width = 10). The variance was comparable between *Nimble* and *Cutometer* measurements. The corrected $k_{corr}^{R0}$ error is the lowest, which is in line with the result of Fig 9 and associated with the general homogenization of these values over different locations.

#### 3.3.3 Offset

**Fig 11:**
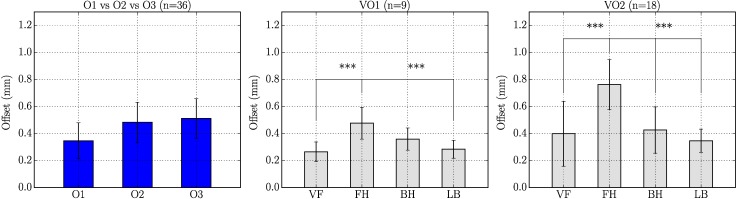
Mean and standard deviation of Offset values in mm (measured by the *Cutometer* software). Left: Mean Offset over all locations for each observer (O1, O2 and O3). Middle/Right: Mean Offset over all observers for each location (VF, FH, BH and LB) separated for two subjects (VO1 and VO2).

The results of the previous sections highlight the differences between corrected and non-corrected stiffness values obtained with the *Cutometer*. Accounting for the initial Offset reduces measurement variability and leads to a general agreement between *k*^*nimble*^ and $k_{corr}^{cuto}$. Importantly, the correlation between observers is significantly improved by the correction and the relative ranking of locations is also affected by the correction of stiffness values. This indicates a possible systematic difference in the Offset generated by different observers and at the different locations. A corresponding analysis is proposed in Fig 11 which reports average Offset values and STD for the three observer (left plot) or the four locations of two different participants (VO1 and VO2), middle and right plot. Mean values of Offset ranges between 0.2 and 0.8 mm, which is relatively large when compared to the reference distance of 1 mm of the *Nimble*. Observer 1 seems to exert lower forces, while O2 and O3 are similar. For both volunteers, the highest Offset was observed for the forehead. These values are significantly higher than for most other locations and this might explain the apparent high stiffness obtained with *k*^*cuto*^ for the forehead (Fig 8). These results indicate that systematic differences of Offset values might be associated with measurements by one specific observer or at one specific body location.

## 4. Discussion

Based on extensive experience with aspiration experiments and, in particular, with the use of the *Cutometer* for skin characterization, the fundamental idea motivating this research was to realize a suction device allowing to minimize the contact force and the influence of patient movements during the measurements. As discussed by Bonaparte et al. [23], the contact force constitutes a relevant influential factor for skin suction experiments. Higher contact force leads to larger initial elevation levels and thus stiffer response. While previous investigations [24,25] analyze the applicability of stable stands to apply controlled contact forces, a novel light-weight suction device is introduced in this study for which such auxiliary measures are not required. The present work aimed to describe the characteristics of the *Nimble* but also to quantify its ability to characterize the mechanical response for different skin locations.

The quantitative assessment of the ability to differentiate among all four tested locations in two subjects was provided through the intraclass correlation coefficient, which was very large for the stiffness measured by the *Nimble* (*k*^*nimble*^, ICC_VO1_ = 0.88, ICC_VO2_ = 0.94). These results confirm that the new technique might improve the reliability of skin characterization.

The mean stiffness determined with the *Nimble* does not correlate with the one obtained using the *Cutometer*. This discrepancy is attributed to the systematic effects associated with the contact force applied during *Cutometer* measurements. On the one hand, the data reported in Fig 5 convincingly demonstrate the effect of the contact force on the *Cutometer* reading. On the other hand, Fig 11 shows that the contact force (resulting in a corresponding Offset value) can differ systematically between body locations and observers.

Supported by corresponding finite element simulations, a correction procedure is proposed to compensate, to some extent, the influence of the contact force. Application of the correction procedure leads to a realignment of stiffness values obtained with the *Cutometer* and *Nimble* at 1 mm tissue elevation (*k*^*nimble*^ and *k*^*cuto*^). In addition, after correction, the scatter associated with *Cutometer* parameters was considerably reduced and the inter-observer Pearson’s coefficient improved for $k_{corr}^{cuto}$ at levels similar to *k*^*nimble*^. These results confirm both, the validity of the *Nimble* measurements, as well as the effectiveness of the correction. Since the Offset value is normally available in *Cutometer* measurements, it is recommended to apply the proposed correction in order to improve the reliability of skin characterization. It should be noted, however, that the reliability of the proposed correction depends on the mechanical behavior of the substrate to be characterized, as shown by the results on Ecoflex, S2 Table.

Another hypothesis analyzed in the present work was that a lower level of deformation would improve the ability of the suction experiment to distinguish between skin locations. In fact, due to the strong strain stiffening response of the dermal layers, at larger values modest changes of elevation occur despite significant increase in suction pressure, thus reducing the sensitivity of the measurements. This hypothesis was confirmed in that the *k*^*R*0^ parameters (corresponding to mean elevations of 1.5 mm) was less able to distinguish between skin locations as compared with *k*^*nimble*^ (1 mm), despite similar values in inter- and intra-observer variability. As a consequence, it is expected that application of lower values of maximum suction pressure (e.g. 200 mbar) would improve the results of a *Cutometer* based characterization.

The *Cutometer* is a sophisticated measuring system providing recording of Pressure - Elevation curves from each measurement. These data can be analyzed to extract information on the elastic and dissipative behavior of superficial tissues and characterize the time and history dependence of the mechanical response. This set of parameters represents a far richer outcome compared to the single parameter provided by the *Nimble*. As indicated in reference [35], additional information on the viscoelastic and viscoplastic properties of skin might be obtained with the *Nimble* through measurements at different suction rates (*p*_*cl*_ for slow and fast suction) or through cyclic application of suction processes (evolution of *p*_*cl*_ in consecutive measurements). The absence of a continuous reading of elevation associated with the imposed pressure rate remains, however, a limitation of the new device. This was the price to pay for an ultralight solution.

Despite this limitation, the *Nimble* might provide significant advantages for clinical applications. The results of the present study indicate that the level of intra- and inter-rater reliability of the *Nimble* is comparable to the one of the *Cutometer*. On the other hand, the ICC of the *Nimble* indicates a better ability to distinguish between locations (***k***^***nimble***^: ICC_VO1_ = 0.88 and ICC_VO2_ = 0.94, ***k***^***R*0**^: ICC_VO1_ = 0.64 and ICC_VO2_ = 0.87, corrected $k_{corr}^{cuto}$: ICC_VO1_ = 0.58 and ICC_VO2_ = 0.83). While this improvement might provide advantages in terms of sensitivity and specificity of the procedure, there are other specific features of the *Nimble*, which could be important for its use in clinical applications: (i) the working principle of the *Nimble* is inherently safer than the *Cutometer* and other suction devices prescribing the maximum negative pressure applied on the tissue. In fact, risk of skin rupture is associated with excessive deformation and the displacement controlled measurement of the *Nimble* allows to directly limit the imposed deformation. The present study applied a maximum displacement of 1 mm but preliminary measurements performed in preparation of clinical studies on scar tissue indicated that the same level of reliability and repeatability can be achieved with a *Nimble* version limiting the maximum displacement to 0.5 mm, which is appropriate for less compliant tissues [6,42]. While the maximum negative pressure of the *Cutometer* can be adjusted for every measurement, it is not possible to predict the level of tissue displacement associated with a specific pressure level, and thus to ensure a priory that a certain level of deformation will not be exceeded. The pressure level limiting the displacement to 0.5 mm for a compliant tissue would lead to non-measurable (too small) deformations at a stiff skin location. (ii) The *Nimble* can be used as a disposable device: the suction probe, silicone tubes and air filters are inexpensive and can be replaced for every measurement. In this way, any cross contamination can be excluded. Other suction devices, such as the *Cutometer*, have to be disinfected after each application, but cannot be sterilized, thus preventing their use on subjects for which the presence of multi resistant bacteria cannot be excluded. (iii) The lightweight of the *Nimble* makes it less sensitive to patient’s movements during the measurement, which is an important advantage for clinical applications. As reported in supplementary information S4 Fig a comparison of the devices was performed in terms of their sensitivity to movements. The parameters measured by the *Cutometer* showed significant differences compared with static measurements, whereas the effect for the *Nimble* was insignificant.

The low variability and high interobserver correlation reported in section 3.3.2 indicate that reliable and objective readings might be obtained independently of the body location or observer. This might facilitate the application of the *Nimble* for a wide range of pathologies and patients, including pediatric patients. In line with these encouraging results, ethical permission was recently obtained for the realization of two explorative clinical studies: one on the early detection of skin modification associated with scleroderma, and the other for the assessment of scar maturation after skin transplants or spontaneously healed wounds on children affected by scars from large burn or scald wounds.

## 5. Conclusions

A novel technique is introduced for suction experiments on skin. The advantages of realizing an ultralight suction probe are demonstrated through a systematic comparison with the *Cutometer*, i.e. the suction device most widely applied for skin characterization. As an inherent uncertainty associated with a hand held device, the influence of the contact force is analyzed in detail and a correction procedure is proposed to reduce its effect on measurement outcomes. *Cutometer* and *Nimble* measurements at different body locations were consistent after correction confirming both, the reliability of the new device and the effectiveness of the correction procedure. As an important byproduct of the present study, the correction is recommended for future applications of the *Cutometer*. Reduced scatter and tissue interrogation at lower level of deformation led to a better ability of the *Nimble* to distinguish between the different skin locations, leading to a high value of the intraclass correlation coefficient and high inter-observer correlation.

As a consequence of the design simplifications leading to the ultralight suction probe, no continuous measurement of tissue elevation is provided by the *Nimble*. While this limits the possibility of characterizing the viscoelastic and viscoplastic properties of skin, the single parameter obtained with the *Nimble* test might be adequate for clinical applications. Its safety, simplicity, and reliability motivates future studies to quantify the diagnostic relevance of the *Nimble* measurement as well as its usefulness in the management of scar treatment in large wounds.

## Supporting information

S1 Fig Vertical measurement set-up. (A) Schematic of the measurement set-up: a holder keeps the *Cutometer*/*Nimble* probe in place and the specimen is placed on top of the probe. (B) The specimen weight leads to an initial protrusion (Δ) of the tissue, penetrating the *Cutometer*/*Nimble* probe.
(TIF)

S2 Fig Finite element meshes for simulation of the *Nimble* and the *Cutometer* aspiration. The instruments are considered as rigid bodies. The skin tissue consists of dermis (t = 1.2mm), subcutaneous tissue (t = 1.5 mm) and underlying muscle tissue (t = 7.3 mm); the Rubin-Bodner constitutive model[40] was implemented for each layer. The same model was used for simulation of measurements on elastomer and all layers had same properties in this case (Neo-Hookean hyperelastic, C_10_ = 0.01167 MPa). The contact interaction between rigid body and skin uses a friction coefficient which was varied in a range μ = 0.0 ÷ 1.0 in a parametric study. For this analysis, the initial contact force (and thus initial protrusion) was zero for all calculations. Quadrilateral axisymmetric elements were used and the mesh size was optimized to ensure adequate discretization of the regions characterized by large stress and strain gradients.
(TIF)

S3 Fig Suction measurements on elastomer. Mean and standard deviation of closing pressure measured by *Nimble* (lightgrey) and *Cutometer* (grey). Significant difference is indicated with p < 0.001 between the devices for m1 and p < 0.01 for m2. Measurements were performed on elastomer (Ecoflex 0030)–specimen weight m1 = 1.5 g and m2 = 50 g.
(TIF)

S4 Fig Influence of patient movement on suction measurements. *Nimble* (blue) and *Cutometer* (red) were tested for patient movement. Data shown are mean and standard deviations of n = 3 repetitions of measurements on human volar forearm in static condition (no movement), rotation (orbital shaker, horizontal circular motion with approximately 1.5 cm/sec), and vertical movement (vertical stage, up-down motion with approximately 5 cm/sec). Rotational and vertical movements led to significantly different closing pressure results in *Cutometer* measurements. Significance level p < 0.05 (*) and p < 0.001 (***).
(TIF)

S1 Table Rubin-Bodner[40] constitutive model parameters for each soft tissue considered in the FE model. The values are based on those presented in [39] and adapted in order to match the present measurements on skin.
(TIF)

S2 Table Analysis of the proposed correction scheme for *Cutometer* measurements and calculations on elastomer model. Results of FE calculations with enforced initial deformation (corresponding to contact forces of 50 g and 100 g) are compared for closing pressure values before and after corrections.
(TIF)

S3 TableMeasurements on volunteers.Reported are data of repeated measurements by three observers (O1, O2 and O3) on two subjects (VO1 and VO2) at four body locations (VF, FH, BH and LB). Data include the Offset Δ, the maximum elevation R0 and the closing pressure $p_{cl}^{cuto}$ of *Cutometer*, and $p_{cl}^{nimble}$ of *Nimble*.(TIF)
