# Supplementary figures and images for: A novel ultra-light suction device for mechanical characterization of skin
Source: PLoS One. 2018 Aug 8;13(8):e0201440. doi: 10.1371/journal.pone.0201440 (PMC6082559; doi:10.1371/journal.pone.0201440)

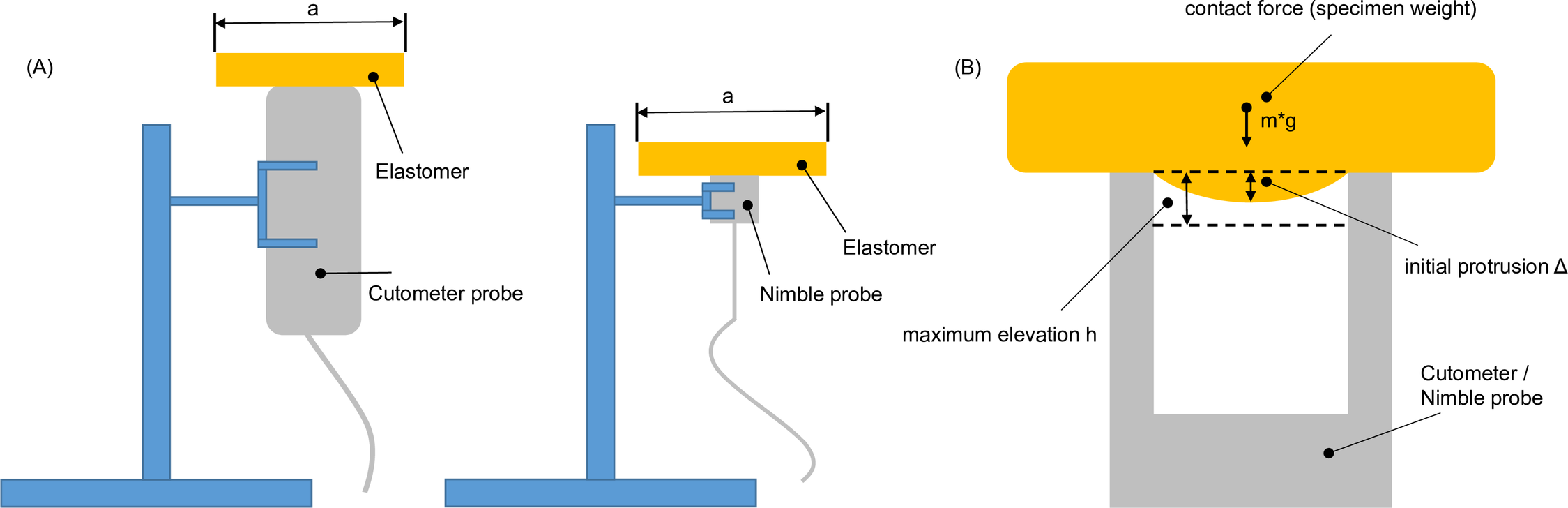

Supplement: S1 Fig — (A) Schematic of the measurement set-up: a holder keeps the Cutometer/Nimble probe in place and the specimen is placed on top of the probe. (B) The specimen weight leads to an initial protrusion (Δ) of the tissue, penetrating the Cutometer/Nimble probe. (TIF) [file pone.0201440.s001.tif]

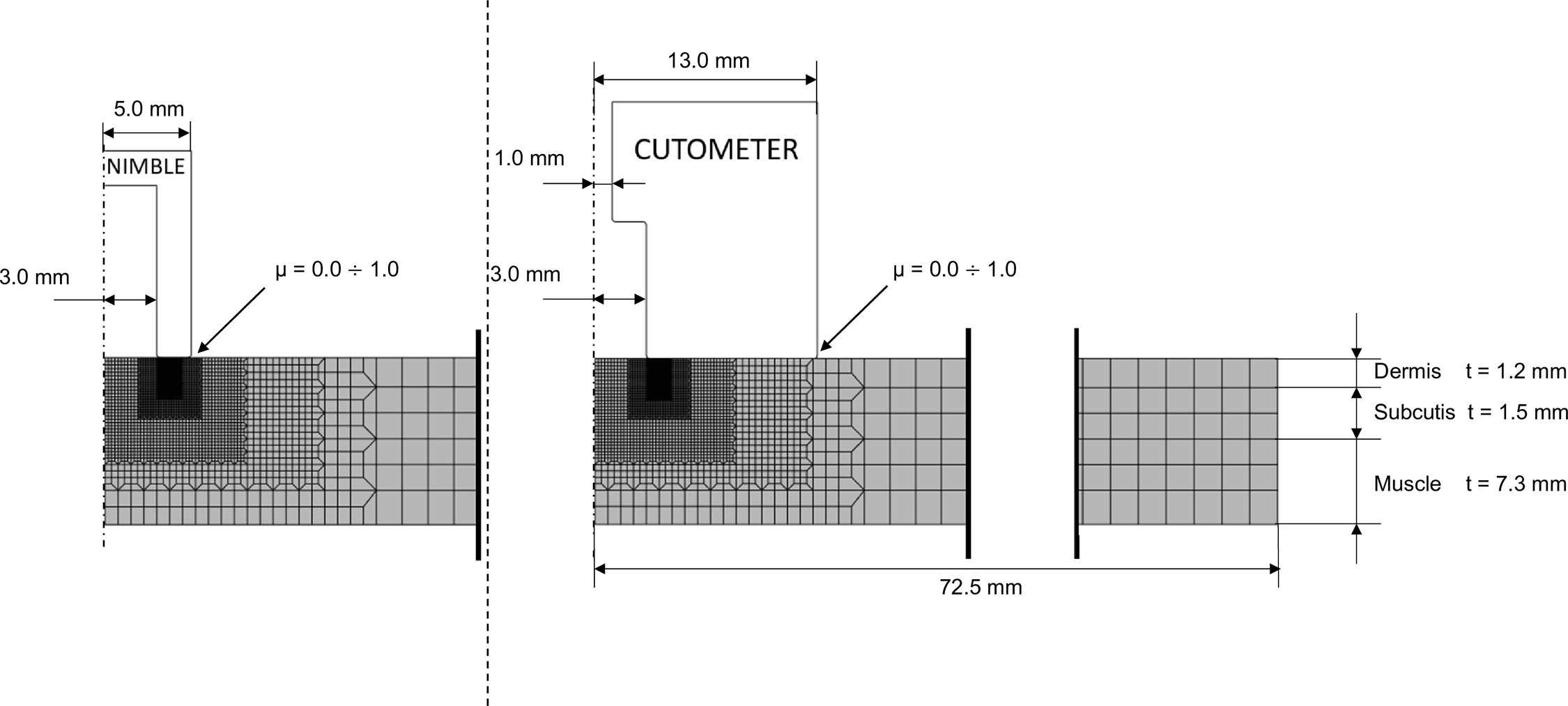

Supplement: S2 Fig — The instruments are considered as rigid bodies. The skin tissue consists of dermis (t = 1.2mm), subcutaneous tissue (t = 1.5 mm) and underlying muscle tissue (t = 7.3 mm); the Rubin-Bodner constitutive model[40] was implemented for each layer. The same model was used for simulation of measurements on elastomer and all layers had same properties in this case (Neo-Hookean hyperelastic, C10 = 0.01167 MPa). The contact interaction between rigid body and skin uses a friction coefficient which was varied in a range μ = 0.0 ÷ 1.0 in a parametric study. For this analysis, the initial contact force (and thus initial protrusion) was zero for all calculations. Quadrilateral axisymmetric elements were used and the mesh size was optimized to ensure adequate discretization of the regions characterized by large stress and strain gradients. (TIF) [file pone.0201440.s002.tif]

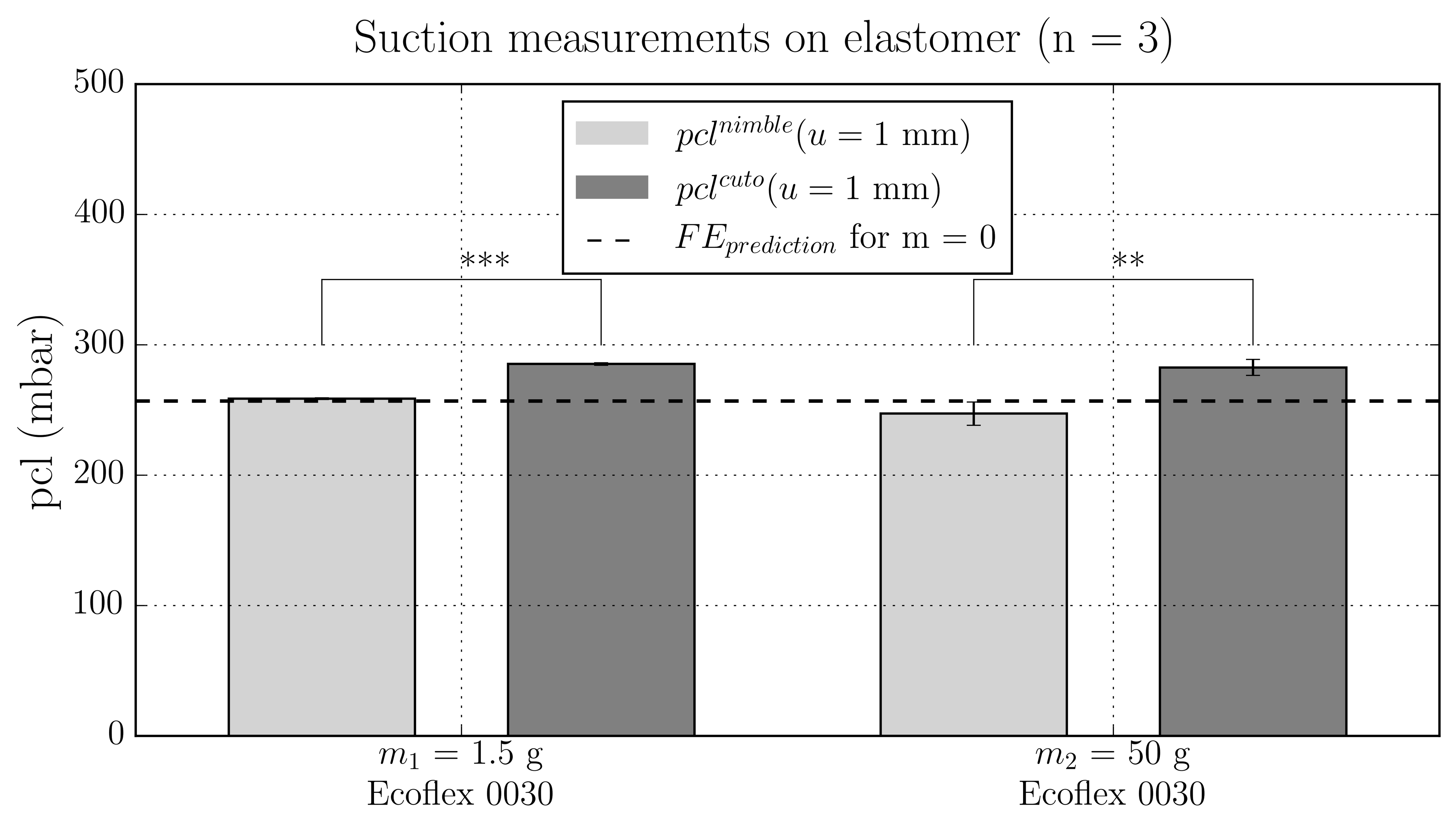

Supplement: S3 Fig — Mean and standard deviation of closing pressure measured by Nimble (lightgrey) and Cutometer (grey). Significant difference is indicated with p < 0.001 between the devices for m1 and p < 0.01 for m2. Measurements were performed on elastomer (Ecoflex 0030)–specimen weight m1 = 1.5 g and m2 = 50 g. (TIF) [file pone.0201440.s003.tif]

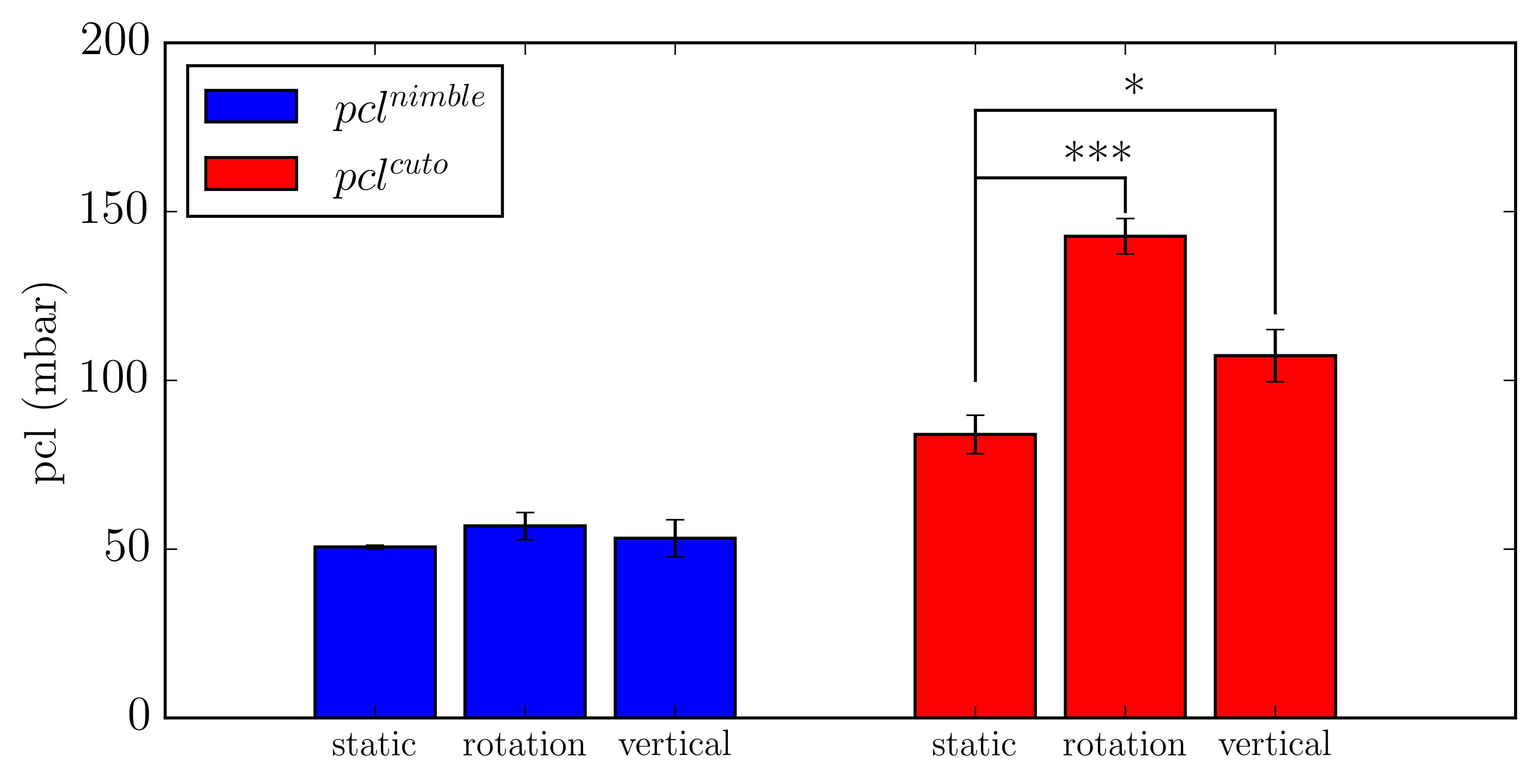

Supplement: S4 Fig — Nimble (blue) and Cutometer (red) were tested for patient movement. Data shown are mean and standard deviations of n = 3 repetitions of measurements on human volar forearm in static condition (no movement), rotation (orbital shaker, horizontal circular motion with approximately 1.5 cm/sec), and vertical movement (vertical stage, up-down motion with approximately 5 cm/sec). Rotational and vertical movements led to significantly different closing pressure results in Cutometer measurements. Significance level p < 0.05 (*) and p < 0.001 (***). (TIF) [file pone.0201440.s004.tif]

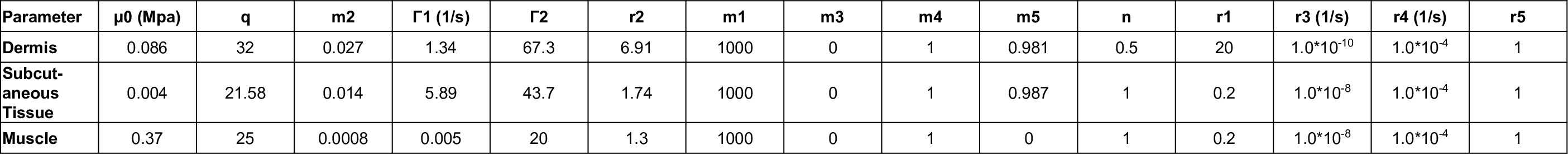

Supplement: S1 Table — The values are based on those presented in [39] and adapted in order to match the present measurements on skin. (TIF) [file pone.0201440.s005.tif]

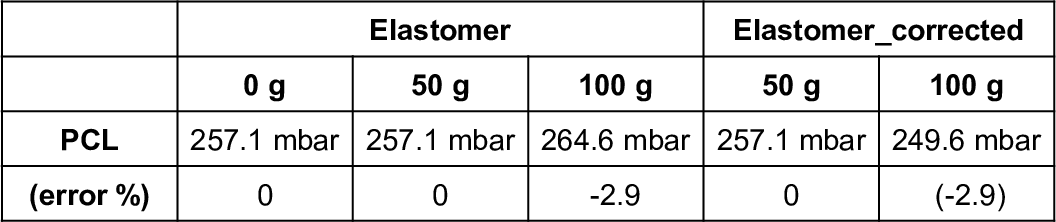

Supplement: S2 Table — Results of FE calculations with enforced initial deformation (corresponding to contact forces of 50 g and 100 g) are compared for closing pressure values before and after corrections. (TIF) [file pone.0201440.s006.tif]

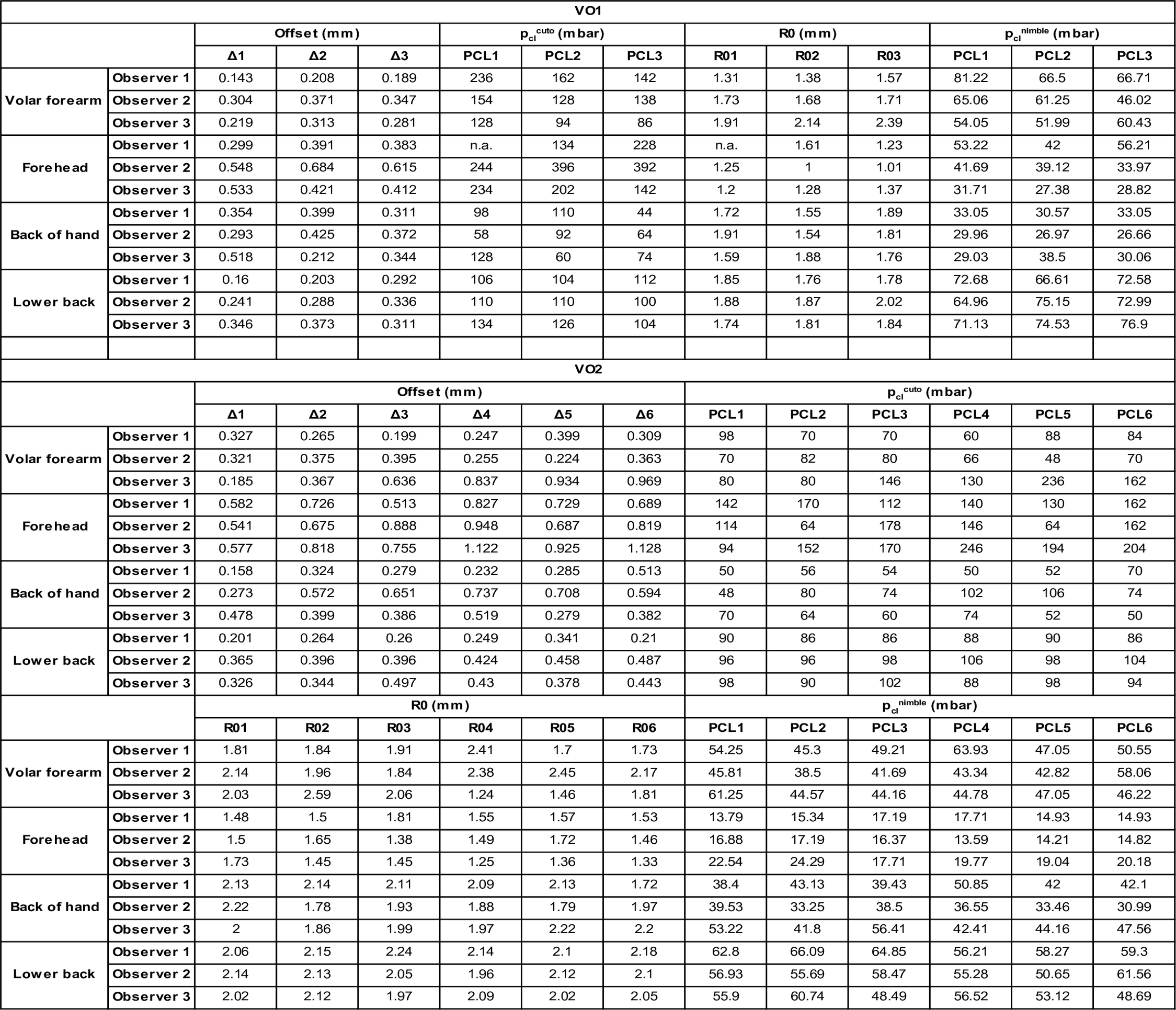

Supplement: S3 Table — Reported are data of repeated measurements by three observers (O1, O2 and O3) on two subjects (VO1 and VO2) at four body locations (VF, FH, BH and LB). Data include the Offset Δ, the maximum elevation R0 and the closing pressure pclcuto of Cutometer, and pclnimble of Nimble. (TIF) [file pone.0201440.s007.tif]
